# A Study of Fever Remedies*Kansas State Homœopathic Society, 1896.

**Published:** 1896-07

**Authors:** E. W. Boardman

**Affiliations:** Parsons, Kansas


### A STUDY OF FEVER REMEDIES.*

E. W. Boardman, M. D., Parsons, Kansas.

Fevers are characterized by rigors, heat of the skin, quick pulse, languor, lassitude, followed by perspiration. A fever may be idiopathic, without any local cause, or symptomatic of some local irritation.

It may be paroxysmal or continued in nature, contagious or traumatic in origin.

There are periods of exacerbation and of remission, days of crisis and many phenomena developed. With theories and explanations we are not dealing in this paper, but shall study for a few moments how to adapt a remedy homœopathically to an individual case.

We must study the history, probable cause, fever symptoms, and concomitant symptoms in endeavoring to find a remedy which will check all developments. It is not sufficient to know that typhoid fever will frequently call for Baptisia, or that lung fever often requires Aconite. In prescribing homœopathically we must set aside the names of diseases and give the remedy indicated, regardless of precedent, even if it is unheard of in the disease under treatment.

---

* Kansas State Homœopathic Society, 1896.

But while this is a true principle of homœopathie prescribing, the intelligent prescriber will consider the history, etiology, and pathology of his case. If he finds a history of exposure to extreme cold, producing a chill, followed by high fever of a certain type which has continued but a few hours before he was called, the knowledge of these facts, taken in conjunction with certain febrile and concomitant symptoms will all assist him in his selection of a remedy. If, again, he finds that the patient has been failing gradually in a general way, becoming progressively weaker, apathetic, with some headache, anorexia, that he has been with persons sick with typhoid fever, or in the same locality, and that for several days before consulting the physician he has had fever, the prescriber will think of an entirely different remedy or class of remedies from that which he thought of in the former case, because such an etiology and pathology will not produce symptoms similar to those of the former case.

Aconite will not cure a fever which depends upon an alteration in the blood, such as is produced by malarial or typhoid poison or bacteria or ptomaines, because these poisons, in changing the normal constituents and relations of blood corpuscles and blood plasma, will not produce symptoms to which Aconite is the similia. For this reason it appears proper that we should study a few remedies, any one of which is likely to be needed when we are considering a given disease, rather than to waste time studying numerous remedies which cannot, in the nature of the case, be indicated.

A comprehensive study of homœopathic materia medica will harmonize the etiological, pathological, and symptomatic indications of our remedies, and strengthen the faith of the earnest student.

In a study of so limited length, however, it will be necessary to confine the attention mainly to symptomatic indications, and to a few of the remedies most often indicated in pyrexia, not, however, forgetting that symptoms may be induced by fever calling for any remedy in the known materia medica.

Probably all will admit that Aconite is the king of fever remedies, a time-honored polychrest, a sheet-anchor in Homœ-

opathy. But if Aconite is prescribed merely because the pulse is abnormally quickened and the temperature is rising, we may be disappointed in its action. A paper of large dimensions might be written on the pulse indications alone. What a difference between the quick, full, and hard pulse of Aconite and the full, flowing, but soft pulse of Gelsemium, or the extremely rapid and tumultuous pulse of Veratrum-viride, or the quick, weak, and irregular pulse of Arsenicum, or the small, hard, rapid, and irregular pulse of China, or the quick, full, and soft pulse of Baptisia, or the throbbing, forcible, and frequent pulse of Belladonna, any one of which may be combined with high temperature. In the examination of a patient with fever, the pulse and temperature are usually noted first, and right here may often be found the keynote, which, when backed up by other symptoms, leads to the selection of the simillimum.

Next the condition of the tongue may be investigated, not merely to learn whether the fever is gastric, inflammatory, typhoid, or intermittent in type, but to ascertain what remedy corresponds to it. We find that in simple fever or in the first access of inflammatory fever with a high temperature, the quick, full, and hard pulse, and a white tongue, Aconite is indicated. Where we find a bounding pulse with a dry red tongue, or with white centre and red edges, Belladonna is likely to come in well. Veratrum-viride has nearly the reverse of Belladonna, a red streak down the centre instead of red edges, and the margins of the tongue yellow. A tongue with red tip and furred sides, or dry and brown, combined with a rapid, weak, and irregular pulse suggests Arsenicum. Baptisia has a yellow tongue shading to dark brown in the centre, accompanied with sordes on the teeth, Bryonia has a thick white coating, China yellow or dirty white, and Gelsemium a thick flabby tongue with a yellowish white coating.

Rhus-tox. has a dry and cracked tongue with a very red tip.

The variation in respiration, as well as the ratio of respiration to the pulse is also marked in these few remedies. Normally there should be three and one-half to four pulse beats to every complete respiration. In Aconite we find a quick and superficial respiration, but with the pulse-respiration ratio maintained, often accompanied with hacking or croupy cough, and with lancinating pains through the chest. Arsenicum has difficult breathing, rather irregular, sometimes an asthmatic struggle for breath, several very shallow inspirations, with occasionally a deep sigh to fill the lungs. Belladonna has short, hurried, and anxious breathing, frequently with tickling in the larynx. When Bryonia is indicated, we find a disposition to breathe deeply, but with great pain and considerable rapidity, even one respiration to three pulse beats. In Gelsemium we find very slow breathing, with rapid pulse; and even more promptly under Veratum-viride, where the ratio may be reduced as low as one respiration to seven or eight pulse beats.

The mentality of fever is also of great importance, in differentiating between remedies. I need only suggest the anxious restlessness of Aconite with great fear of death; the scattered feeling of Baptisia, with inability to get the parts together; the wild delirium with horrible visions, and disposition to jump out of bed, which characterizes the Belladonna patient; the morose, ill-humored anxiety, with ravings about his business affairs, and desire to go home, which constitutes the mentality of the Bryonia patient. Just as familiar are the sluggish stupor, and inability to fix the attention, of the Gelsemium patient; the anxious fear and extreme restlessness, with dread of death, of Arsenicum; the confusion of ideas and dislike to all exertion produced by taking Quinine, and consequently indicating China when not so produced. Rhus-tox. has great restlessness, or a low mild delirium with stupefaction, and Veratrum-viride has a mentality as greatly excited as is its circulation.

Many lay great stress upon the exact hour of aggravation, but it is a clinical fact that nearly all fever patients are worse toward and during the night, and febrile symptoms are also aggravated by any excitement, at whatever hour. According to text-books, Aconite is worse in the evening and night; Arsenicum from 1 to 3 A. M; Belladonna after 3 P. M., and after midnight; China in the morning or evening and every other day; Gelsemium in the evening; Rhus-tox. after midnight; Veratrum-viride in the evening.

But few remedies have been considered in this paper, but probably the fever symptoms of a majority of cases may be pretty well covered by these polychrests. If physicians would more carefully study the old and well-proven remedies in our materia medica, they would often find it unnecessary to fall back upon the refuse of a *gas factory* for anti-pyretics, even to administer new remedies lauded commercially by pharmacists without proper proving.

Our noble ancestors, medically speaking, have performed cures innumerable with the polychrests. Why not we?

